# Calcium Transient and Sodium-Calcium Exchange Current in Human versus Rabbit Sinoatrial Node Pacemaker Cells

**DOI:** 10.1155/2013/507872

**Published:** 2013-02-24

**Authors:** Arie O. Verkerk, Marcel M. G. J. van Borren, Ronald Wilders

**Affiliations:** ^1^Department of Anatomy, Embryology and Physiology, Academic Medical Center, University of Amsterdam, Meibergdreef 15, 1105 AZ Amsterdam, The Netherlands; ^2^Laboratory of Clinical Chemistry and Hematology, Jeroen Bosch Hospital, Henri Dunantstraat 1, 5223 GZ 's-Hertogenbosch, The Netherlands

## Abstract

There is an ongoing debate on the mechanism underlying the pacemaker activity of sinoatrial node (SAN) cells, focusing on the relative importance of the “membrane clock” and the “Ca^2+^ clock” in the generation of the small net membrane current that depolarizes the cell towards the action potential threshold. Specifically, the debate centers around the question whether the membrane clock-driven hyperpolarization-activated current, *I*_*f*_, which is also known as the “funny current” or “pacemaker current,” or the Ca^2+^ clock-driven sodium-calcium exchange current, *I*_NaCa_, is the main contributor to diastolic depolarization. In our contribution to this journal's “Special Issue on Cardiac Electrophysiology,” we present a numerical reconstruction of *I*_*f*_ and *I*_NaCa_ in isolated rabbit and human SAN pacemaker cells based on experimental data on action potentials, *I*_*f*_, and intracellular calcium concentration ([Ca^2+^]_*i*_) that we have acquired from these cells. The human SAN pacemaker cells have a smaller *I*_*f*_, a weaker [Ca^2+^]_*i*_ transient, and a smaller *I*_NaCa_ than the rabbit cells. However, when compared to the diastolic net membrane current, *I*_NaCa_ is of similar size in human and rabbit SAN pacemaker cells, whereas *I*_*f*_ is smaller in human than in rabbit cells.

## 1. Introduction

Animal studies have demonstrated that pacemaker activity of the sinoatrial node (SAN) is controlled by a complex system of “clocks” composed of voltage-dependent sarcolemmal currents—designated the “membrane clock,” “voltage clock,” or “ion channel clock”—and tightly coupled sarcoplasmic reticulum (SR) Ca^2+^ cycling molecules together with the electrogenic sodium-calcium exchanger, named the “Ca^2+^ clock” [1, 2]. There is an ongoing debate on the relative importance of the “membrane clock” and the “Ca^2+^ clock” in the generation of the small net membrane current underlying the spontaneous diastolic depolarization that drives the cell towards its action potential threshold [3–7]. This debate centers around the contribution of the hyperpolarization-activated current *I*_*f*_, also known as “funny current” or “pacemaker current,” as a member of the membrane clock, and the sodium-calcium exchange current *I*_NaCa_, resulting from the electrogenic sodium-calcium exchange process and thus driven by the Ca^2+^ clock.

In contrast with the data collected in animal studies, the mechanism of SAN pacemaker activity in man is virtually unexplored. In a comprehensive study, Chandler et al. [8] characterized the “molecular architecture” of the human SAN based on messenger RNA (mRNA) levels of 120 ion channels and related proteins, and they concluded that the expression pattern was appropriate to explain pacemaking. They observed a prominent expression of HCN4 and, to a lesser extent, HCN1, which are both subunits of the *I*_*f*_ channel. We actually recorded *I*_*f*_ in voltage clamp experiments on single pacemaker cells isolated from the SAN of a patient who underwent SAN excision [9]. From these cells, we also acquired spontaneous action potentials, which showed a clear diastolic depolarization phase resulting in an intrinsic cycle length of ≈830 ms (72 beats/min). In addition, our voltage clamp experiments revealed the presence of a fast large inward current with characteristics of the Na^+^ current, *I*_Na_ [10].

Clinical data also point to a role for *I*_*f*_ and *I*_Na_ in human SAN pacemaker activity. Mutations in *HCN4* and *SCN5A*, encoding pore-forming subunits of the *I*_*f*_ and *I*_Na_ channel, respectively, have been linked to familial sick sinus syndrome (see [11, 12] and primary references cited therein), thus suggesting that *I*_*f*_ and *I*_Na_ indeed contribute to human SAN pacemaker activity. A further clinical indication regarding the role of a specific ion current in human SAN pacemaker activity involves the slowly activating delayed rectifier K^+^ current (*I*_Ks_) and comes from patients who suffer from the long-QT syndrome type 1 and carry a loss-of-function mutation in the *KCNQ1* (*KvLQT1*) gene, encoding the pore-forming *α*-subunit of the *I*_Ks_ channel. These patients have close-to-normal heart rates in rest [13], but their ability to increase heart rate during exercise is seriously impaired [14].

The aforementioned experimental and clinical observations all point to a role for important components of the “membrane clock” in human SAN pacemaker activity. There are also some clinical data in support of a contribution of the “Ca^2+^ clock” through patients with catecholaminergic polymorphic ventricular tachycardia (CPVT), who have an impaired Ca^2+^ clock due to mutations in the *RYR2* gene (CPVT1) or the *CASQ2* gene (CPVT2), encoding the cardiac ryanodine receptor isoform 2 (RyR2, responsible for Ca^2+^ release from the SR) and the cardiac calsequestrin isoform 2 protein (calsequestrin-2, responsible for calcium buffering in the SR), respectively [15]. Leenhardt et al. [16] observed a marked sinus bradycardia in a group of 21 nongenotyped CPVT patients. Sumitomo et al. [17] also reported sinus bradycardia in their group of 29 nongenotyped CPVT patients. At the time of the reports by Leenhardt et al. [16] and Sumitomo et al. [17], CPVT had not yet been associated with the *RYR2* and *CASQ2* genes. More recently, Postma et al. [18, 19] found a marked sinus bradycardia in both CPVT1 [18] and CPVT2 [19] patients.

Chandler et al. [8] observed various Ca^2+^-handling proteins in the human SAN, albeit less abundant than in the surrounding right atrium, including NCX1 (responsible for sodium-calcium exchange in the heart), RyR2, and SERCA2a (responsible for Ca^2+^ uptake by the SR). They constructed a mathematical model of a human SAN cell by modification of the Courtemanche et al. [20] model of a human atrial myocyte, scaling ion current densities on the basis of the relative mRNA expression level in SAN and atrium and introducing the T-type Ca^2+^ current and *I*_*f*_. Specifically, *I*_NaCa_ was scaled down to 74% of its value in the Courtemanche et al. [20] model. Recently, Allah et al. [21] published a study in which they used the same experimental approach as Chandler et al. [8] to determine the expression of ion channels and Ca^2+^ handling proteins in the SAN, right atrium, and left ventricle of the neonate and adult rabbit. From their Figure 6 [21], it appears that the mRNA expression level of the *NCX1* gene in the adult rabbit SAN is ≈78% and ≈69% of that in right atrium and left ventricle, respectively.

When we recorded action potentials from isolated human SAN pacemaker cells and carried out voltage clamp experiments [9], we also acquired some data on the intracellular free Ca^2+^ concentration ([Ca^2+^]_*i*_) of human SAN pacemaker cells using the fluorescent Ca^2+^ indicator Indo-1. In the present study, we use these thus far unpublished experimental data in a numerical reconstruction of *I*_NaCa_ in a human SAN pacemaker cell. The thus obtained *I*_NaCa_ is compared to the net membrane current (*I*_net_) and to *I*_*f*_, which are also obtained through a numerical reconstruction. In addition, we present data on *I*_*f*_, *I*_NaCa_, and *I*_net_ in rabbit SAN pacemaker cells, thus allowing a comparison of these currents between rabbit and human SAN pacemaker cells.

## 2. Materials and Methods

### 2.1. Cell Preparations

Single SAN cells were isolated by an enzymatic dissociation procedure as described previously [22] from New Zealand White rabbits and from a patient who underwent SAN excision because of inappropriate sinus tachycardias originating from the SAN region (see [9] for clinical details). In either case, cells were stored at room temperature for at least 45 min in modified Kraft-Brühe (KB) solution before they were put into a recording chamber on the stage of an inverted microscope and superfused with modified Tyrode's solution at 36 ± 0.2°C. KB solution contained (in mM) KCl 85, K_2_HPO_4_ 30, MgSO_4_ 5.0, glucose 20, pyruvic acid 5.0, creatine 5.0, taurine 30, *β*-hydroxybutyric acid 5.0, succinic acid 5.0, BSA 1%, and Na_2_ATP 2.0; pH was set to 6.9 with KOH. Modified Tyrode's solution contained (in mM) NaCl 140, KCl 5.4, CaCl_2_ 1.8, MgCl_2_ 1.0, glucose 5.5, and HEPES 5.0; pH was set to 7.4 with NaOH. Spindle and elongated spindle-like cells displaying regular contractions were selected for measurements.

### 2.2. Cytosolic Ca^2+^ Measurements

Cytosolic Ca^2+^ concentration ([Ca^2+^]_*i*_) was measured in Indo-1 loaded cells as described previously [23]. In brief, cells were loaded with 5 *µ*M of the fluorescent dye Indo-1-AM (Molecular Probes, Eugene, OR, USA) for 10 min at room temperature in KB solution and subsequently superfused with modified Tyrode's solution for 15 min at 36 ± 0.2°C to remove excess indicator and allow full deesterification. A rectangular adjustable slit was used to select a single cell and to reduce background fluorescence. Dual wavelength emission of Indo-1 upon excitation at 340 nm was recorded at 405–440 and 505–540 nm using photomultiplier tubes, and, after correction for background fluorescence, free [Ca^2+^]_*i*_ was calculated as described by van Borren et al. [23].

[Ca^2+^]_*i*_ transients were characterized by the minimum diastolic [Ca^2+^]_*i*_ (MDC), their amplitude (TA), their maximum rate of rise (d[Ca^2+^]_*i*_/d*t*_max_), their duration measured at 20, 50, and 90% decay (TD_20_, TD_50_, and TD_90_, resp.), and their frequency. Parameter values obtained from 10 consecutive [Ca^2+^]_*i*_ transients were averaged.

### 2.3. Action Potential Measurements

Action potentials from rabbit and human SAN cells were recorded with the amphotericin-perforated and conventional whole-cell configuration of the patch-clamp technique, respectively, using an Axopatch 200B patch-clamp amplifier (Molecular Devices Corporation, Sunnyvale, CA, USA). For recording from rabbit SAN cells, pipettes (borosilicate glass; resistance 2–5 MΩ) were filled with solution containing (in mM) K-gluconate 120, KCl 20, NaCl 5, amphotericin B 0.22, NMDGCl (N-methyl-D-glucammonium chloride) 10, and HEPES 10; pH was set to 7.2 with KOH. For recording from human SAN cells, patch pipettes contained (in mM) K-gluconate 125, KCl 20, NaCl 5, MgCl_2_ 1, MgATP 5, and HEPES 10; pH was set to 7.2 with KOH.

Action potentials were characterized by their frequency, their duration at 20, 50, and 90% repolarization (APD_20_, APD_50_, and APD_90_, resp.), maximum diastolic potential (MDP), action potential amplitude (APA), maximum upstroke velocity (d*V*_*m*_/d*t*_max_), and diastolic depolarization rate measured over the 100 ms time interval starting at MDP + 1 mV (DDR_100_). Parameter values obtained from 10 consecutive action potentials were averaged. All membrane potential values were corrected for the calculated liquid junction potential.

### 2.4. Numerical Reconstruction of Hyperpolarization-Activated Current

For the numerical reconstruction of both rabbit and human *I*_*f*_, we used a first-order Hodgkin and Huxley [24] type kinetic scheme. Accordingly, *I*_*f*_ is given by

(1) $$\begin{array}{c} I_{f}=y\times g_{f}\times \left(V_{m}-E_{f}\right), \end{array}$$

in which *g*_*f*_ is the fully activated *I*_*f*_ conductance, *V*_*m*_ is the membrane potential, *E*_*f*_ is the *I*_*f*_ reversal potential, and the gating variable *y*, with 0 ≤ *y* ≤ 1, obeys the first-order differential equation

(2) $$\begin{array}{c} \frac{\text{d}y}{\text{d}t}=\alpha \times \left(1-y\right)-\beta \times y, \end{array}$$

with voltage-dependent rate constants *α* and *β*, or, equivalently,

(3) $$\begin{array}{c} \frac{\text{d}y}{\text{d}t}=\frac{\left(y_{\infty }-y\right)}{\tau }\;, \end{array}$$

with the steady-state activation *y*_*∞*_ and time constant *τ* given by

(4) $$\begin{array}{c} y_{\infty }=\frac{\alpha }{\left(\alpha +\beta \right)}, \end{array}$$

(5) $$\begin{array}{c} \tau =\frac{1}{\left(\alpha +\beta \right)}\;. \end{array}$$

For rabbit *I*_*f*_, we used the model by Dokos et al. [25], who based their equations on the comprehensive experimental study on rabbit *I*_*f*_ by van Ginneken and Giles [26], arriving at

(6) $$\begin{array}{c} \alpha =\frac{0.36\times \left(V_{m}+137.8\right)}{\left\{\exp\left[0.066\times \left(V_{m}+137.8\right)\right]-1\right\}},\\ \beta =\frac{0.1\times \left(V_{m}+76.3\right)}{\left\{1-\exp\left[-0.21\times \left(V_{m}+76.3\right)\right]\right\}}, \end{array}$$

for the rate constants *α* and *β*, both expressed in s^−1^. In our computations, we used a scaling factor of 0.71665 for the time constant *τ* (5) as a correction factor for the temperature of 30–33°C in the experiments of van Ginneken and Giles [26]. This correction factor was adopted from the SAN cell models by Kurata et al. [27] and Maltsev and Lakatta [28]. With the bath and pipette Na^+^ and K^+^ concentrations listed in Sections 2.1 and 2.3, the reversal potential of the Dokos et al. [25] model amounts to −21.0 mV. For the fully activated *I*_*f*_ conductance, we used a value of 0.218 pS/pF, based on the mean values of 12.0 nS and 55 pF for the fully activated conductance and membrane capacitance, respectively, reported by van Ginneken and Giles [26].

For human *I*_*f*_, we used the model that we developed on the basis of our voltage clamp data on human SAN cells [9]. In this model [29, 30], *y*_*∞*_ and *τ* (in ms) are given by

(7) $$\begin{array}{c} y_{\infty }=0.01329+\frac{0.99921}{\left\{1+\exp\left[\left(V_{m}+97.134\right)/8.1752\right]\right\}},\\ \;\;\;\text{if}\;\;V_{m}<-80\;\text{mV},\\ y_{\infty }=0.0002501\times \exp\left(\frac{-V_{m}}{12.861}\right)\;\text{if}\;\;V_{m}\geq -80\;\;\text{mV}, \end{array}$$

(8) $$\begin{array}{c} \tau =1000\times \{0.36\;\;\;\;\;\;\;\;\;\;\;\;\;\;\\ \;\times \frac{\left(V_{m}+148.8\right)}{\left[\exp\left(0.066\;\times \left(V_{m}+148.8\right)\right)-1\right]}+0.1\\ {\times \frac{\left(V_{m}+87.3\right)}{\left[1-\exp\left(-0.21\times \left(V_{m}+87.3\right)\right)\right]}\}}^{-1}-54, \end{array}$$

respectively. The fully activated *I*_*f*_ conductance *g*_*f*_ and the *I*_*f*_ reversal potential *E*_*f*_ were set to 0.075 nS/pF and −22 mV, respectively, in accordance with the fully activated *I*_*f*_ conductance of 75.2 ± 3.8 pS/pF (mean ± SEM, *n* = 3) and *I*_*f*_ reversal potential of −22.1 mV ± 2.4 mV (mean ± SEM, *n* = 3) determined experimentally [9].

The numerical reconstruction was carried out on an Intel Xeon based workstation using Compaq Visual Fortran 6.6C and employing a simple and efficient Euler-type integration scheme with a time step of 10 *µ*s. Simulations were run for a sufficiently long time to achieve steady-state conditions.

### 2.5. Numerical Reconstruction of Sodium-Calcium Exchange Current

For the numerical reconstruction of human *I*_NaCa_ on the basis of the recorded data on *V*_*m*_ and [Ca^2+^]_*i*_ (combined voltage and calcium clamp [31]), we adopted the *I*_NaCa_ formulation of the Courtemanche et al. [20] model for a human atrial myocyte, with *I*_NaCa_ scaled down to 74% of its control value. We thus followed the approach by Chandler et al. [8] in their construction of a mathematical model of a human SAN cell by modification of the Courtemanche et al. [20] model, as set out in the Introduction.

We used a similar approach for the numerical reconstruction of rabbit *I*_NaCa_ on the basis of the acquired data on *V*_*m*_ and [Ca^2+^]_*i*_. In this case, we adopted the *I*_NaCa_ formulation of the Lindblad et al. [32] model for a rabbit atrial myocyte. However, the original *I*_NaCa_ was scaled down to 78% of its control value, based on the mRNA data by Allah et al. [21], as detailed in the Introduction.

The Courtemanche et al. [20] and Lindblad et al. [32] equations not only require values for *V*_*m*_ and [Ca^2+^]_*i*_, but also for [Ca^2+^]_*e*_, [Na^+^]_*e*_ and [Na^+^]_*i*_, which denote the extracellular Ca^2+^ concentration, the extracellular Na^+^ concentration, and the intracellular Na^+^ concentration, respectively. For these ion concentrations, we used the bath and pipette solutions listed in Sections 2.1 and 2.3.

### 2.6. Statistics

Data are presented as mean ± SEM. Comparisons between groups were made using an unpaired *t*-test. The level of significance was set at *P* < 0.05.

## 3. Results

For the present study, we first characterized the action potentials and [Ca^2+^]_*i*_ transients that we recorded from single rabbit and human SAN pacemaker cells. Next, we used our experimental data for a numerical reconstruction of the hyperpolarization-activated current and the sodium-calcium exchange current associated with the recorded action potentials and [Ca^2+^]_*i*_ transients, thus allowing a comparison of these currents between rabbit and human SAN pacemaker cells.

### 3.1. Rabbit and Human Action Potentials

**Figure 1:**
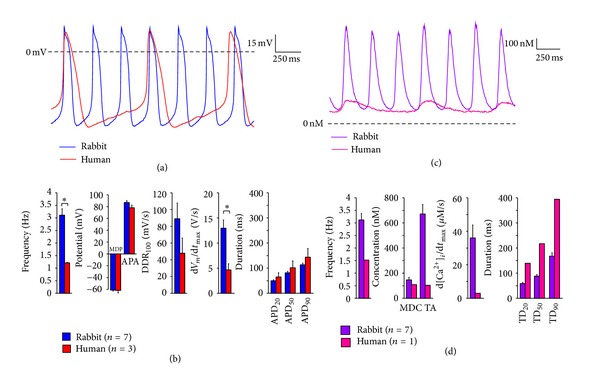
Spontaneous electrical activity of rabbit and human sinoatrial node (SAN) pacemaker cells. (a) and (b) Typical examples (a) and average characteristics (b) of action potentials. (c) and (d) Typical examples (c) and average characteristics (d) of the intracellular free Ca^2+^ concentration ([Ca^2+^]_*i*_). Data are mean ± SEM; *n* = number of cells; MDP = maximum diastolic potential; APA = action potential amplitude; DDR_100_ = diastolic depolarization rate over the 100 ms time interval starting at MDP + 1 mV; d*V*_*m*_/d*t*_max_ = maximal upstroke velocity; APD_20_, APD_50_, and APD_90_ = action potential duration at 20, 50, and 90% repolarization; MDC = minimum diastolic [Ca^2+^]_*i*_; TA = [Ca^2+^]_*i*_ transient amplitude; d[Ca^2+^]_*i*_/d*t*_max_ = maximum rate of rise of [Ca^2+^]_*i*_ transient; TD_20_, TD_50_, and TD90 = [Ca^2+^]_*i*_ transient duration at 20, 50, and 90% decay. *P < 0.05.

Figure 1(a) shows typical examples of the spontaneous action potentials of rabbit and human SAN pacemaker cells, recorded with the patch-clamp technique. Maximum diastolic potential (MDP) and action potential amplitude (APA) are remarkably similar, whereas the beating frequency is strikingly different, as confirmed by the mean value of 3.11 ± 0.24 Hz for the 7 rabbit cells versus 1.21 ± 0.02 Hz for the 3 human cells (*P* < 0.05) and illustrated in Figure 1(b).

Neither the action potential durations, for example, the APD_90_ of 144 ± 35 versus 113 ± 6 ms, nor the diastolic depolarization rate of 49 ± 18 versus 89 ± 19 mV/s differ significantly between human and rabbit SAN cells, which is likely due to the low number of cells of human origin. Nevertheless, from Figure 1(a), it is indicative that the longer cycle length of the human cells (828 ± 15 versus 337 ± 35 ms) is importantly due to longer diastolic depolarization rather than longer action potential duration. A further difference is found in the maximum rate of rise of the action potential, which is considerably larger in the rabbit cells (13.0 ± 1.6 versus 4.6 ± 1.2 V/s, *P* < 0.05; Figure 1(b)).

### 3.2. Rabbit and Human [Ca^2+^]_*i*_ Transients

In the 7 rabbit cells of Figure 1(b), we simultaneously recorded the membrane potential, using the perforated patch-clamp technique, and the [Ca^2+^]_*i*_ transient, using the fluorescent Ca^2+^ indicator Indo-1. A typical example of such [Ca^2+^]_*i*_ transient, recorded from the same cell and during the same period of time as used for Figure 1(a), is provided in Figure 1(c). Also shown in Figure 1(c) is the [Ca^2+^]_*i*_ transient that we were able to record from a single human SAN cell. Further attempts to acquire the [Ca^2+^]_*i*_ transient of human cells resulted in unstable recordings. Of note, no action potentials are available from the cell that we recorded the [Ca^2+^]_*i*_ transient from. So, in contrast with the rabbit data, the human data of Figures 1(a) and 1(c) are not simultaneously collected and are not from the same cell.

The minimum diastolic [Ca^2+^]_*i*_ level of the human cell is similar to that of the rabbit cells, but otherwise the [Ca^2+^]_*i*_ transients are widely different, with a smaller amplitude, longer duration, and smaller rate of rise in case of the human cell, as illustrated in Figure 1(d), in which the characteristics of the [Ca^2+^]_*i*_ transient of the human cell are compared to those of the mean characteristics in the 7 rabbit cells.

**Figure 2:**
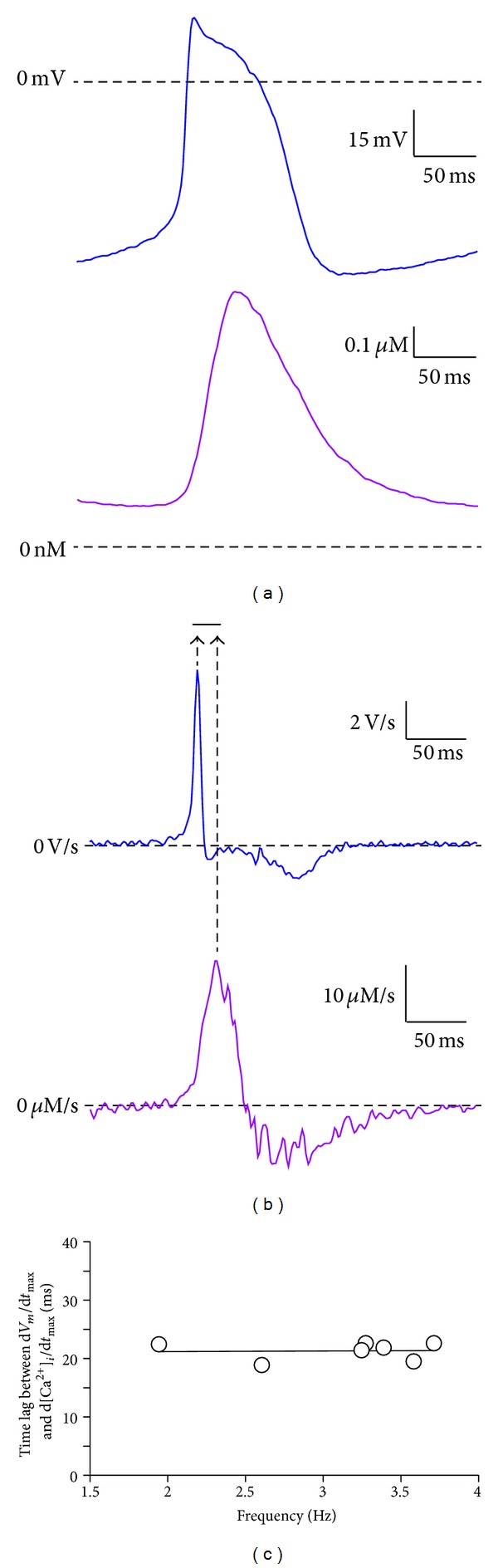
(a), Simultaneously recorded action potential (top) and [Ca^2+^]_*i*_ transient (bottom) of a rabbit SAN cell. (b) Time derivatives of the action potential (top) and [Ca^2+^]_*i*_ transient (bottom) traces shown in panel (a). Arrows indicate the timing of the maximum rate of change in membrane potential (d*V*_*m*_/d*t*_max_) and intracellular Ca^2+^ concentration (d[Ca^2+^]_*i*_/d*t*_max_). Horizontal bar indicates the time lag of ≈20 ms. (c) Time lag between the occurrence of d*V*_*m*_/d*t*_max_ and d[Ca^2+^]_*i*_/d*t*_max_ versus beating frequency of the 7 cells measured. Solid line is the linear regression line (slope 0.1 ± 1.1; *R* = 0.042). Note the absence of an appreciable frequency dependence of the time lag.

As illustrated in Figure 2(a) for the simultaneously recorded action potential (top) and [Ca^2+^]_*i*_ transient (bottom) of a rabbit SAN cell, the maximum rate of rise of the [Ca^2+^]_*i*_ transient, as determined from the time derivative of the [Ca^2+^]_*i*_ transient signal (Figure 2(b), bottom), occurs with a time lag relative to the maximum rate of rise of the associated action potential, as determined from the time derivative of the *V*_*m*_ signal (Figure 2(b), top). This time lag between the occurrence of d*V*_*m*_/d*t*_max_ and d[Ca^2+^]_*i*_/d*t*_max_ was determined for each of the 7 rabbit SAN cells tested and amounted to 21.3 ± 0.6 ms. The time lag ranged between 19 and 23 ms and showed no appreciable frequency dependence, as illustrated in Figure 2(c), in which the time lag is plotted versus the spontaneous beating frequency.

### 3.3. Numerical Reconstruction of Membrane Ionic Currents

Next, we carried out a numerical reconstruction of the hyperpolarization-activated current (*I*_*f*_) and the sodium-calcium exchange current (*I*_NaCa_) of rabbit and human SAN pacemaker cells based on the experimental data on their action potentials and [Ca^2+^]_*i*_ transients presented in Figures 1 and 2.

#### 3.3.1. Experimental Data

**Figure 3:**
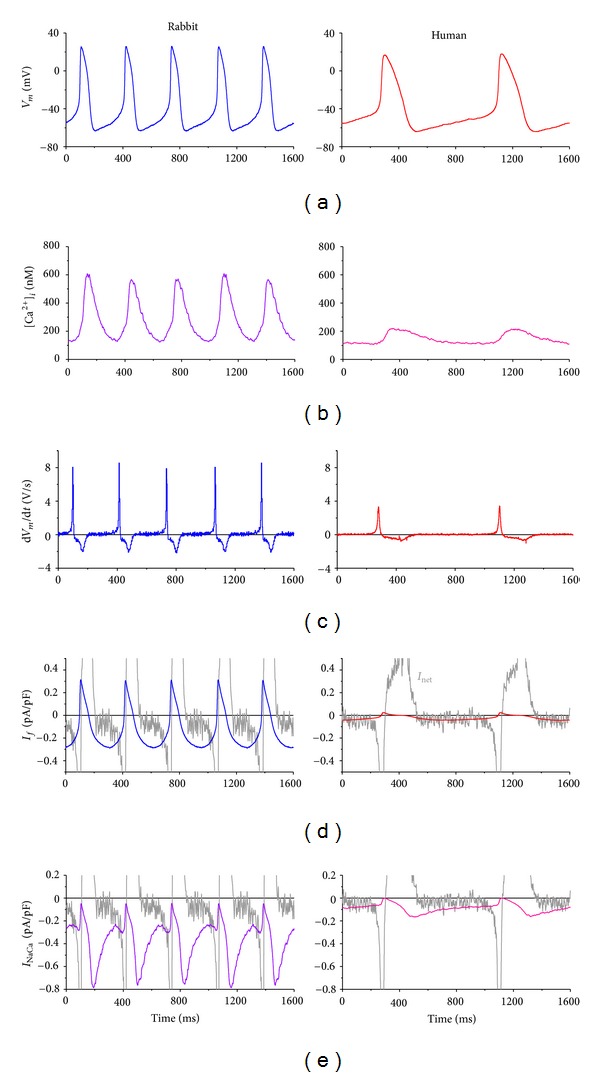
Action potential and calcium transient recordings from rabbit (left) and human (right) SA nodal myocytes and associated numerical reconstructions of membrane currents. (a) Recorded membrane potential *V*_*m*_. (b) Recorded intracellular calcium concentration ([Ca^2+^]_*i*_). (c) Time derivative of *V*_*m*_ (d*V*_*m*_/d*t*). (d) Reconstructed hyperpolarization-activated inward current (*I*_*f*_). The noisy trace in gray is the net membrane current (*I*_net_) computed from *I*_net_ = −*C*_*m*_ × d*V*_*m*_/d*t*, where *C*_*m*_ denotes membrane capacitance. (e) Reconstructed sodium-calcium exchange current (*I*_NaCa_), with the net membrane current in gray.

For the reconstruction of rabbit membrane ionic currents, we used a 1-s simultaneous recording of *V*_*m*_ and [Ca^2+^]_*i*_ of one of the 7 rabbit cells that we digitized at 10 kHz through linear interpolation of the acquired data points. This 1-s recording contained three action potentials and associated [Ca^2+^]_*i*_ transients and was turned into a continuous signal by repeating the 1-s signal. Because of the repetition of the 1-s signal, the fourth and fifth action potentials of Figure 3(a) (left) are identical to the first and second, respectively. The same holds for the associated [Ca^2+^]_*i*_ transients, which are shown in Figure 3(b) (left).

A similar approach, now using a ≈1.6 s time frame, was used in case of the human cell data. However, because data on *V*_*m*_ and [Ca^2+^]_*i*_ were not simultaneously collected, we selected two consecutive action potentials with cycle lengths of 791 and 822 ms—human SAN pacemaker cells show a beat-to-beat fluctuation in cycle length as do rabbit cells [33]—and two consecutive [Ca^2+^]_*i*_ transients that, by coincidence, had identical cycle lengths. Furthermore, for the timing of the two signals, it was assumed that the time lag between the occurrence of d*V*_*m*_/d*t*_max_ and d[Ca^2+^]_*i*_/d*t*_max_ was 21 ms, as observed in the rabbit cells (Figure 2(c)). The resulting *V*_*m*_ and [Ca^2+^]_*i*_ data are shown in the right panels of Figures 3(a) and 3(b).

#### 3.3.2. Numerical Reconstruction of Net Membrane Current

As a reference for *I*_*f*_ and *I*_NaCa_, we first determined the net membrane current (*I*_net_) in both rabbit and human cells. Because *I*_net_ = −*C*_*m*_ × d*V*_*m*_/d*t*, where *C*_*m*_ denotes membrane capacitance, the current density of *I*_net_ is identical to −d*V*_*m*_/d*t* and thus readily follows from the time derivative of the action potential, which is shown in Figure 3(c) for the action potentials of Figure 3(a). In the diastolic voltage range, *I*_net_ is a small inward current with a density of ≈0.1 and ≈0.05 pA/pF in rabbit and human, respectively (Figures 3(d) and 3(e), gray traces), in line with the mean diastolic depolarization rate of 89 ± 19 and 49 ± 18 mV/s, respectively (Figure 1(b)). Figure 3(c) also confirms the data of Figure 1(b) regarding the smaller d*V*_*m*_/d*t*_max_ in human versus rabbit SAN cells.

#### 3.3.3. Numerical Reconstruction of Hyperpolarization-Activated Current

The action potentials of Figure 3(a) were applied as a voltage clamp signal in order to reconstruct the hyperpolarization-activated current *I*_*f*_, as detailed in Section 2.4. The resulting *I*_*f*_ traces are shown in Figure 3(d). There is an almost 7-fold difference in diastolic *I*_*f*_ amplitude between rabbit and human, which becomes less pronounced when compared to the net membrane current, *I*_net_, in each of the cell types. In rabbit, diastolic *I*_*f*_ is 2-3 times *I*_net_, whereas diastolic *I*_*f*_ and *I*_net_ are of similar size in human.

#### 3.3.4. Numerical Reconstruction of Sodium-Calcium Exchange Current

The *V*_*m*_ and [Ca^2+^]_*i*_ data of Figures 3(a) and 3(b) were applied as a combined voltage and calcium clamp signal in order to reconstruct the sodium-calcium exchange current *I*_NaCa_ in human and rabbit SAN cells, as detailed in Section 2.5. The resulting *I*_NaCa_ is smaller in amplitude in human than in rabbit (Figure 3(e)). However, in either case, the mid-diastolic *I*_NaCa_ amplitude is roughly twice that of *I*_net_. The end-diastolic amplitude of *I*_NaCa_ at −45 mV is ≈0.07 pA/pF in human, which is ≈25% of the value of ≈0.28 pA/pF in rabbit.

#### 3.3.5. Charge Carried by Individual Currents

**Figure 4:**
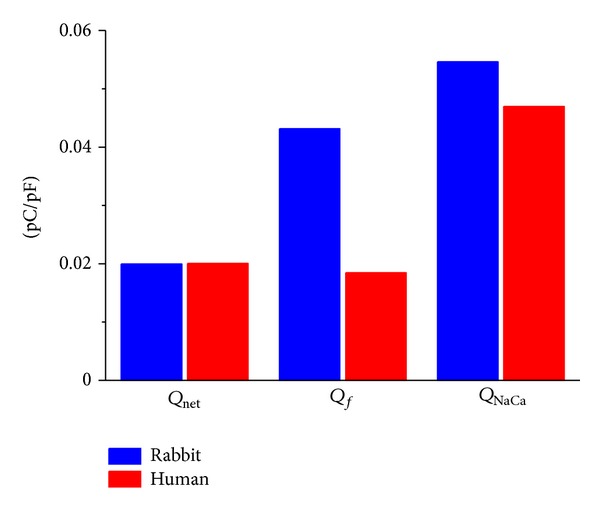
Charge carried by net membrane current (*Q*_net_), reconstructed hyperpolarization-activated inward current (*Q*_*f*_), and reconstructed sodium-calcium exchange current (*Q*_NaCa_) during 20 mV spontaneous depolarization from MDP + 1 mV of the rabbit and human SA nodal action potentials of Figure 3.

From the current traces of Figures 3(d) and 3(e), one can compute the contribution to diastolic depolarization in terms of charge carried by *I*_net_, *I*_*f*_, and *I*_NaCa_ through integration of each of these currents over time. We carried out such computation for the 20 mV spontaneous depolarization from MDP + 1 mV of the rabbit and human action potentials of Figure 3(a). As shown in Figure 4 (left bars), the thus computed charge carried by *I*_net_(*Q*_net_) amounts to 0.020 pC/pF for both rabbit and human, as expected from the 20 mV depolarization, which is equivalent to a charge flow of 0.02 pC/pF. The charge carried by *I*_*f*_(*Q*_*f*_) is 0.043 and 0.018 pC/pF for rabbit and human, respectively, whereas that carried by *I*_NaCa_(*Q*_NaCa_) amounts to 0.055 and 0.047 pC/pF, respectively (Figure 4, middle and right bars).

## 4. Discussion

In the present study, we first characterized the action potentials and [Ca^2+^]_*i*_ transients that we recorded from single rabbit and human SAN pacemaker cells. Next, we used our experimental data for a numerical reconstruction of the hyperpolarization-activated current and the sodium-calcium exchange current associated with the recorded action potentials and [Ca^2+^]_*i*_ transients.

### 4.1. Experimental Data

Human SAN pacemaker cells have a lower beating frequency and a lower maximum upstroke velocity than rabbit SAN pacemaker cells (Figures 1(a) and 1(b)). Also, they have a considerably weaker [Ca^2+^]_*i*_ transient (Figures 1(c) and 1(d)), which may, at least in part, be a frequency effect, as observed in rabbit cells [23]. The longer cycle length of the human cells is largely due to a longer diastolic phase (Figures 1(a) and 1(b)). In this respect, the recorded action potentials differ from those that Chandler et al. [8] computed by turning the Courtemanche et al. [20] human atrial cell model into a human SAN pacemaker cell model. On the other hand, the range of the computed calcium signal (approximately 150–250 nM; Figure 8 of Chandler et al. [8]) compares reasonably well with the experimentally observed range (roughly 110–220 nM; Figures 1(c) and 1(d)).

As an important caveat, it should be noted that our data on human cells have been collected from only a small number of cells, all from a single patient who underwent SAN excision because of tachycardias [9]. Stable action potential recordings were obtained from three cells and a stable calcium transient recording from only one cell. Furthermore, although almost identical experimental methods were employed, the use of the perforated-patch configuration of the patch-clamp technique in case of the rabbit cells and the conventional whole-cell configuration in case of the human cells may have introduced deviations.

### 4.2. Numerical Reconstructions

In our numerical reconstruction of rabbit *I*_*f*_, we used the Dokos et al. [25] equations based on a reanalysis of the data by van Ginneken and Giles [26] rather than the equations provided by van Ginneken and Giles [26] themselves. In the latter equations, the *I*_*f*_ deactivation rate is erroneously overestimated, as set out in detail by Dokos et al. [25]. As a result, the reconstructed *I*_*f*_ of Figure 3(d) (left) is larger than in rabbit SAN cell models that employ the van Ginneken and Giles equations, such as the models by Kurata et al. [27] and Maltsev and Lakatta [28]. Furthermore, the more negative maximum diastolic potential of the recorded action potentials adds to the larger *I*_*f*_. The availability of *I*_*f*_ almost immediately after repolarization and its amplitude larger than that of *I*_net_ are in line with the data from action potential clamp experiments on rabbit SAN cells carried out by Zaza et al. [34]. The reconstructed human *I*_*f*_ trace compares well with the traces that we previously reconstructed for the other two human SAN pacemaker cells from which action potentials are available [12].

In our numerical reconstruction of *I*_NaCa_, we used equations from rabbit and human atrial cell models—properly scaled to account for the reduced mRNA expression of *NCX1* in the SAN versus the atrium, both in human [8] and in rabbit [21]—that allowed the reconstruction of *I*_NaCa_ from the experimentally recorded membrane potential and global cytosolic [Ca^2+^]_*i*_. Today, there are highly detailed models of calcium handling in (rabbit) SAN cells [28, 35], but for a reconstruction of *I*_NaCa_, these would require experimental data on [Ca^2+^]_*i*_ in submembrane spaces rather than global cytosolic [Ca^2+^]_*i*_. In human cells, the total charge carried by *I*_*f*_ during spontaneous diastolic depolarization is similar to that carried by *I*_net_, whereas in rabbit it is approximately twice (Figure 4).

Of note, the diastolic *I*_NaCa_ amplitude at −45 mV of ≈0.28 pA/pF in rabbit (Figure 3(e), left) is identical to the value of 0.28 ± 0.03 pA/pF that Vinogradova et al. [36] observed experimentally. A highly similar amplitude is obtained (data not shown) if *I*_NaCa_ is not reconstructed from the Lindblad et al. [32] equations but from the equations for a ventricular cell provided by Faber and Rudy [37], scaled down to 69% to account for the mRNA data by Allah et al. [21].

Human *I*_NaCa_ is smaller than that of rabbit (Figure 3(e)). This is not only due to the smaller *I*_NaCa_ in the human versus the rabbit atrial cell model from which the equations were adopted for the reconstruction. What also plays a role is that the experimentally recorded [Ca^2+^]_*i*_ is smaller in human than in rabbit at all values of *V*_*m*_. Yet, the total charge carried by *I*_NaCa_ during spontaneous diastolic depolarization is of similar magnitude in rabbit and human (Figure 4).

## 5. Conclusion

Human SAN pacemaker cells have a smaller *I*_*f*_, a weaker [Ca^2+^]_*i*_ transient, and a smaller *I*_NaCa_ than rabbit SAN pacemaker cells. However, when compared to the diastolic net membrane current, *I*_NaCa_ is of similar size in human and rabbit cells, whereas *I*_*f*_ is smaller in human than in rabbit cells.
